# Plant-Based Dairy Alternatives Contribute to a Healthy and Sustainable Diet

**DOI:** 10.3390/nu15153393

**Published:** 2023-07-30

**Authors:** Winston J. Craig, Virginia Messina, Ian Rowland, Angelina Frankowska, Jane Bradbury, Sergiy Smetana, Elphee Medici

**Affiliations:** 1 Center for Nutrition, Healthy Lifestyle, and Disease Prevention, School of Public Health, Loma Linda University, Loma Linda, CA 93254, USA; 2 Nutrition Consultant, Nutrition Matters, Inc., Pittsfield, MA 01201, USA; ginnymessina@gmail.com; 3 Department of Food and Nutritional Sciences, University of Reading, Reading RG6 6DH, UK; i.rowland@reading.ac.uk; 4 Independent Research Consultant, Environmental Sustainability Assessment, Bedford MK45 4BX, UK; angelina.frankowska@cranfield.ac.uk; 5 School of Medicine, Edge Hill University, Ormskirk L39 4QP, UK; jane.bradbury@edgehill.ac.uk; 6 German Institute of Food Technologies (DIL e.v.), 49610 Quakenbrueck, Germany; s.smetana@dil-ev.de; 7 Nutrition & Sustainable Diets Consultant, Nutrilicious Ltd., London N2 0EF, UK; elphee@nutrilicious.co.uk

**Keywords:** sustainability, environmental footprint, dairy alternatives, plant-based drinks, protein, calcium, vitamin B12, iodine, vitamin D, ultra-processed food

## Abstract

Plant-based foods are increasing in popularity as more and more people are concerned about personal and planetary health. The consumption of plant-based dairy alternatives (PBDAs) has assumed a more significant dietary role in populations shifting to more sustainable eating habits. Plant-based drinks (PBDs) made from soya and other legumes have ample protein levels. PBDs that are appropriately fortified have adequate levels of important vitamins and minerals comparable to dairy milk. For the PBDs examined, the greenhouse gas emissions were diminished by 59–71% per 250 mL, and the land use and eutrophication impact was markedly less than the levels displayed by dairy milk. The water usage for the oat and soya drinks, but not rice drinks, was substantially lower compared to dairy milk. When one substitutes the 250 mL serving of dairy milk allowed within the EAT Lancet Planetary Health Diet for a fortified plant-based drink, we found that the nutritional status is not compromised but the environmental footprint is reduced. Combining a nutrient density score with an environmental index can easily lead to a misclassification of food when the full nutrition profile is not utilized or only a selection of environmental factors is used. Many PBDAs have been categorized as ultra-processed foods (UPFs). Such a classification, with the implied adverse nutritional and health associations, is inconsistent with current findings regarding the nutritional quality of such products and may discourage people from transitioning to a plant-based diet with its health and environmental advantages.

## 1. Introduction

Plant-based foods are increasing in popularity with concerns about personal and planetary health. Food-based dietary guidelines (FBDGs) reflect dietary patterns that are associated with a decreased risk of non-communicable diseases (NCDs) and nutrient deficiencies. They may also be associated with more sustainable dietary patterns with an emphasis on plants and plant-based foods and a relatively low contribution of meat and dairy [1,2,3]. Sustainable Healthy Diets are dietary patterns that promote an individual’s health and wellbeing; are accessible, affordable, culturally acceptable, and equitable; have a low environmental impact; and support the preservation of planetary health and biodiversity [4].

Unfortunately, the proportion of the world’s population that meets FBDGs is low [5], hence NCDs make a major contribution globally to premature mortality [6]. There is, therefore, a real need to increase the proportion of the population consuming diets that are closer to the FBDG ideal to improve health and environmental sustainability. Expecting populations to adopt wholly plant-based (vegan) diets is unrealistic and this is reflected in current sustainable dietary recommendations, where the focus is on increasing healthy plant foods, especially plant sources of protein, whilst reducing, rather than avoiding, the consumption of animal-derived protein [2,7,8]. Plant foods such as nuts, legumes and pulses, and cereals, as well as alternative proteins and PBDAs used to produce products that mimic their animal-based counterparts, are one of the fastest-growing sectors within the food industry [9]. This growth is expected to continue to meet increasing demand from meat and dairy consumers who wish to improve the healthfulness and sustainability of their diets [10].

In this paper, we review the role of plant-based dairy alternatives (PBDAs) within current sustainable dietary recommendations, from an environmental, nutritional, and behavioural change perspective. We explore the environmental footprint of PBDAs and the environmental and nutritional consequences of introducing them into the EAT Lancet Planetary Health Diet. This is followed by an extensive review of the literature on PBDAs’ contribution to nutritional adequacy for a European population, including indicators for their nutritional and environmental impacts and ultra-processed classification. And finally, we review how PBDAs could help shift the dietary behaviour of the European population towards more sustainable dietary patterns.

## 2. Materials and Methods

### 2.1. Environmental Impact of Beverages

The environmental impacts of dairy milk and plant-based drinks were calculated from the raw data available from UK researchers [11], and the impact data were normalized to refer to one serving of 250 mL. The environmental impacts for dairy milk and PBDs were based on the European methods of production, rather than using the global averages (see Supplemental Materials). The availability of data for PBDs based on soya, oats, and rice facilitated the comparative analysis with dairy milk. The relative contributions of the different life cycle stages of dairy milk and soya drink were also calculated from that data pool representing the global market [11], since the regional data were unavailable. Relative environmental impacts enable one to identify stages within the supply chain to develop options for improvement. The environmental impact of the nutrient fortification of PBDs was also analysed. The relative impact on greenhouse gas emissions of fortification was calculated following the procedures outlined by Bussa et al. [12]. 

### 2.2. EAT Lancet Analysis

For a European population, we examined the nutritional impact of replacing the 250 mL dairy milk daily allowance within the EAT Lancet Planetary Health Diet (EAT PHD). To ensure we were reflecting European food nutritional values, we undertook a nutritional analysis using European food database software [13] and previously published European low (1.2–1.8%)-fat dairy milk nutritional data [14]. Lower-fat milk was used to reflect the current main usage in Europe and recommendations within food-based dietary guidelines [15]. This formed our EU Baseline EAT PHD. This was further modified to produce two additional variations: replacing 250 mL dairy milk with 250 mL fortified unflavoured soya drink and replacing 250 mL dairy milk with 250 mL fortified unflavoured oat drink. All other components of the EU Baseline remained as originally published. The resulting nutritional changes were recorded. 

To analyse the overall impact of PBDAs on diet, the EAT Lancet Planetary Health Diet was taken as a base scenario [8]. The different food groups of the EAT PHD were linked to the environmental impact data based on the work of Poore and Nemecek [11]. Two different scenarios were evaluated following the nutritional analysis, replacing 250 mL dairy milk with either 250 mL soya drink or 250 mL oat drink, to explore the environmental impact of those dietary changes.

### 2.3. Cost Analysis 

We collected pricing data on the major brands of fortified soya drinks, the private label brands of soya drinks, and the dairy semi-skimmed milk from the leading 5 grocery markets in the UK. This permitted a cost comparison. Details of the analysis appear in an Excel spreadsheet in the Supplementary Materials.

## 3. Results

**Figure 1:**
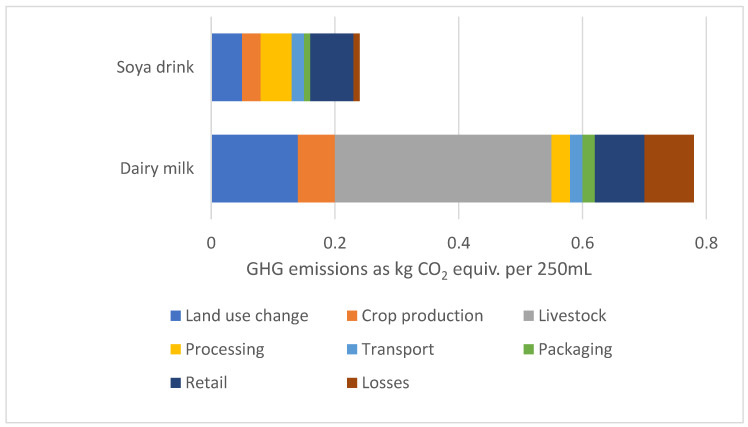
Life cycle impact contribution to the total carbon footprint for 250 mL dairy milk and soya drink—using global average values [11].

**Figure 2:**
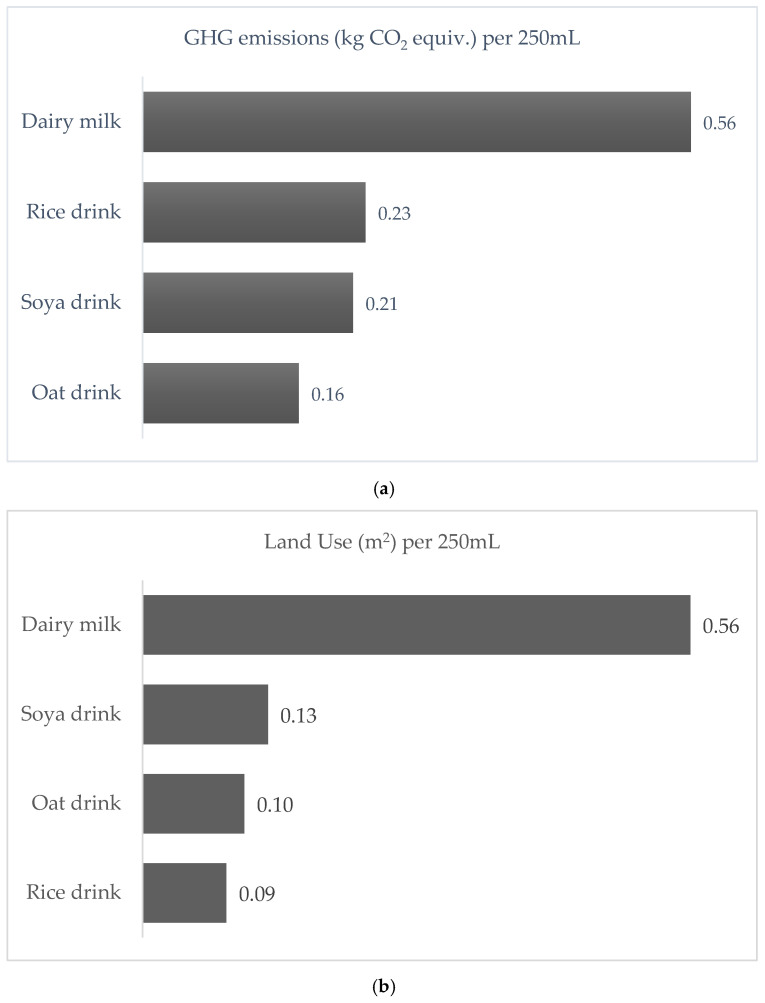

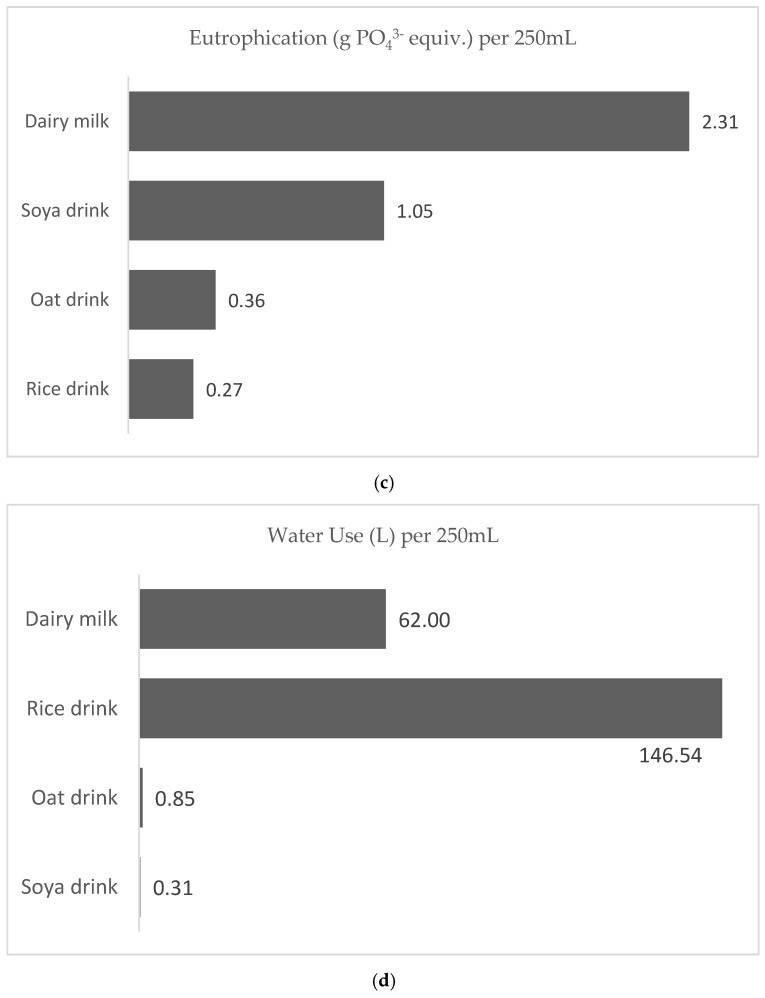
(**a**) Greenhouse gas emissions (GHG) (**b**) Land use (**c**) Eutrophication potential (**d**) Water use of European dairy milk and plant-based drinks per 250 mL [11].

**Figure 3:**
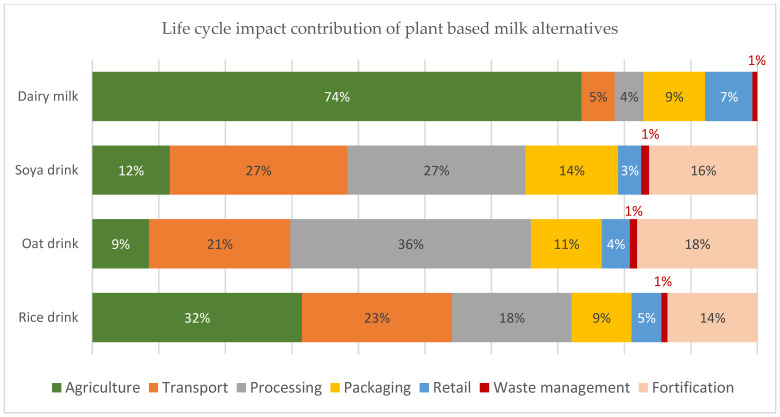
Life cycle impact contribution of European plant-based drinks and dairy milk to the total carbon footprint including the fortification stage in PBDs [12].

The life cycle impact contribution to the total carbon footprint for 250 mL soya drink compared to dairy milk is shown in Figure 1. About 45% of total emissions for dairy milk production come from enteric fermentation, the major contributor. Using the global data, the carbon footprint of dairy is 3.3 times greater than that of soya drinks. Greenhouse gas emissions (GHG), land use, eutrophication potential, and water use for 250 mL dairy milk and plant-based drinks are shown in Figure 2a–d, respectively, using all European values. The carbon footprint for oat, soya, and rice drinks is only 29%, 38%, and 41% that of dairy milk. The land use for rice, oat, and soya drinks amounts to only 16%, 18%, and 23% of the land use for dairy milk. Eutrophication levels for rice, oats, and soya were found to be 12%, 16%, and 45% of that associated with dairy milk. While water use for rice milk was 2.36 times greater than that of dairy milk, water use for oat and soya drinks was only 1.3% and 0.5% that of dairy milk. The life cycle impact contribution of European fortified plant-based drinks and dairy milk towards the total carbon footprint is illustrated in Figure 3. 

The adapted EAT Lancet Planetary Health Diet nutritional values using a European food database compared to the original EAT Lancet analysis using a US food database are shown in Table 1. Changing from a serving of full-cream dairy milk to low-fat dairy milk produced changes of 2% or less in most dietary nutrients. Saturated fat decreased by 9% and the fat-soluble vitamin D decreased by 60%. The nutritional comparison between the EU EAT Lancet Planetary Health Diet using 250 mL low-fat dairy milk versus 250 mL fortified soya or fortified oat-based drink is shown in Table 2. Protein levels decreased by 1% for soya milk and 7% for the rice milk substitution, while calcium levels remained the same with the PBDA substitution. Vitamin D levels increased by 85% and riboflavin levels increased by 5%, while vitamin B12 levels decreased by 5% for both rice and soya substitutions.

## 4. Discussion

### 4.1. Environmental Footprint

The environmental impact of dairy milk varies across different practices and depends on the life span of the cow, the milk yield, and the feed, among other factors [16]. An efficient dairy system where milk yield is maximized and feed optimized will result in a lower impact. On average, European milk is responsible for 2.2 kg CO2e/L from farm to retail which is one of the lowest impacts globally [17]. Most of the emissions (>70%) occur at the farm with enteric fermentation, representing 45% of the total, being the greatest contributor (Figure 1). In comparison, PBDs have a lower carbon footprint compared to dairy milk by 59%, 62%, and 71% for rice, soya, and oat drinks, respectively (Figure 2a) [11].

European dairy milk has the highest impact compared to European PBDs across all environmental indicators except for water use in the production of rice drinks (Figure 2d). This situation exists even though European dairy milk is based on a highly efficient production system. The contrast between environmental indicators for dairy milk and PBDs is more pronounced on the global scene, since the global dairy industry has a much higher impact due to inefficient feeding, low milk yield, and other issues. In the case of water use, European dairy milk requires considerably more water than oat and soya drinks (72-fold and 200-fold, respectively), while European rice drinks require substantially more water (58%) than dairy milk (Figure 2d). This is due to the need in Europe for irrigation water rather than the rainwater used in Asia during the rainy season, which allows Asia to irrigate using less water at higher rice yields. 

European oat drink generates the lowest emissions, followed by soya drink, while rice drink emits the highest GHGs among the dairy-free options (Figure 2a). The rice drink is lowest in the eutrophication impact and occupies the least land but has significantly higher water use (Figure 2b–d). In conclusion, the environmental performance varies for PBDs across different indicators. However, the ecological footprint of dairy milk is the highest for all impacts except for water use, where the water usage for growing rice is more than double the dairy usage [11].

The environmental impact of the fortification stage has been commonly overlooked. Since the fortification of PBDs adds nutritional value, the environmental implications should also be considered. Typically, the fortification stage will account for 14–18% of the total emissions for PBDs (Figure 3). Considering the agricultural, processing and fortification stages, the impact contribution is still lower than the burden of the farming stage of dairy milk. The overall emissions of 1 L of a fortified PBD are still lower by at least 23–39% when considering the fortification stage within the analysis. Moreover, energy use is the main contributor to the fortification processing’s impact, and the decarbonization of energy will reduce these impacts further. Whereas, in the case of dairy milk, the biggest contributor is the methane emissions from the animal’s enteric fermentation [17], and while there are efforts to reduce these emissions, it remains a challenge to lower them significantly.

### 4.2. What Is the Nutritional and Environmental Impact of Introducing PBDs to the EAT Planetary Health Diet

According to the EAT Lancet Planetary Health Diet (EAT PHD), the daily 250 mL full-cream dairy milk allowance accounts for 6% of the calorie and 9% of the protein consumption but contributes 39% to calcium and 48% to vitamin B12 intake [8]. Despite the low calorie and protein contribution, dairy makes up 16% of the total dietary carbon footprint [8]. Replacing dairy milk with PBDs may therefore reduce the overall environmental impact of the diet. 

Table 1 shows the nutritional difference between the US database analysis of the EAT Lancet PHD and our baseline EU EAT PHD analysed using European food database software, switching full dairy for lower-fat dairy to reflect current European dairy consumption and national dietary recommendations. Table 2 shows the nutritional differences between the EU Baseline PHD incorporating 250 mL dairy milk and the two dietary adaptations, replacing the dairy milk with 250 mL fortified soya or oat drink. Consuming soya or oat drink instead of one serving of dairy milk reduces dietary greenhouse gas emissions by 9% and 12%, respectively. The total calcium for the replacement with PBDs is comparable to dairy whilst vitamin D almost doubles, iodine increases by 31%, and vitamin B2 intake modestly increases by 5%. With current vitamin B12 fortification and following the EU EAT PHD with few animal products, this vitamin intake will drop by 5%. However, a higher fortification product or using a vitamin B12 supplement is a good way to avoid low vitamin B12 intake. Protein intake is marginally reduced by 1% and 7%, respectively, when soya or oat drink is substituted. Nevertheless, the diet will still substantially exceed the European dietary reference value for protein by 43–52% (see Table 2). Incorporating PBDs into the diet will not only have benefits for the environment but also will not compromise the nutritional profile of the total diet. 

Replacing one serving of dairy with a fortified soya drink will result in an overall calcium, vitamin B2, and vitamin B12 content that is comparable to consuming just the dairy before the switch. Minor changes in calcium and vitamin content of current diets will not compromise the nutritional quality of the diet of European adults. 

### 4.3. Environmental and Nutritional Indices

Several proposed initiatives aimed to develop an index that combines the environmental impact with the nutritional value of foods. A common limitation of these integrative indices is that they tend to focus on a limited number of factors within the nutritional and environmental domains [18,19,20]. Also, the choice of functional unit (mass, energy, serving size, nutrient density, protein, etc.) to express the result can strongly influence the interpretation and comparability of different foods [21]. Furthermore, the nutrient density, as well as the climate impact of foods, has to be considered with respect to the total diet.

The usefulness of nutrient density scores, in combination with environmental parameters, is limited by the lack of harmonization [22]. There is significant heterogeneity between different nutrient density scores; for example, the choice of nutrients, the number of nutrients included, and the weight attributed to each in the index (nutrients that should be encouraged and/or nutrients to limit), and no or little adaptation to specific population needs or taking into consideration the current level of intakes compared to dietary reference values. Additionally, how these are integrated with environmental assessments highly impacts the interpretation and recommendations of which foods are best to consume [23,24,25,26,27,28,29,30]. The combined indices can be very misleading in the broader context of dietary advice.

More recently, the use of integrated indicators that rely solely on protein quality and carbon footprint has been discussed [24]. These indicators may not be applicable in Western countries where protein intakes typically exceed recommendations. For example, when considering a balanced vegan diet in its entirety, it is well established that all essential amino acids are provided in sufficient quantities to meet requirements, despite several individual plant proteins having a limiting amino acid profile. Furthermore, concentrating on greenhouse gas emissions (GHGe) as a climate impact indicator, while useful, fails to account for the total environmental impact of food systems, as it does not account for factors such as biodiversity loss, eutrophication, land use, and water footprint. Not incorporating the full nutrition profile and the diverse environmental impacts of foods can easily lead one to misclassify food products. Cultural dimensions such as equity and animal welfare and economic dimensions such as affordability are also important elements to capture.

### 4.4. Nutrient Adequacy

#### 4.4.1. Protein

While plant foods contain all the essential amino acids, their amino acid profile varies from one protein to another. When a variety of plant foods are eaten and energy needs are met, the various proteins complement each other [31]. Legumes are a rich source of protein, with soya having a high-quality protein (i.e., quantity, amino acid composition, and bioavailability) equivalent to animal protein [32]. Among the PBDs, those based on pea protein or soya beans have a protein content comparable to dairy milk [33]. Both pea and soya proteins are rated as good-quality proteins by standard methods [34]. 

PBDs made from grains or nuts have considerably lower levels of protein unless they have added soya or pea protein [33]. However, the replacement of dairy milk in European populations with PBDs containing lower protein levels will not compromise one’s protein status since the overall protein intake typically greatly exceeds the requirements [35,36,37,38].

From survey data, we observe that Europeans may consume more than double the protein Population Reference Intake (PRI) for the general population [36]. National dietary surveys of 12 European countries reveal that protein intakes range from 62 to 111 g for adult men and 70 to 130 g for women. This exceeds the PRI of 58 g protein per day for a reference body weight in Europe of 70 kg [39,40]. Replacing two 250 mL servings of milk (containing 8 g protein/serving) with a plant-based drink containing 1 to 3 g of protein/serving would reduce overall protein intake by 10–14 g. Therefore, a daily protein intake of 100 g/d containing two servings of milk would be reduced to an intake of 86–90 g/d by this substitution, which is still above the recommended intake.

Clearly, dairy milk is not essential to ensure an adequate protein intake in European adult populations. The replacement of dairy milk with a plant-based drink with a lower protein content seems very unlikely to result in an inadequate protein intake given the high overall protein consumption of adults and the fact that there are plenty of other protein-rich foods available. Even for European school-aged children, the protein intake has been reported [41,42] to be more than adequate. 

#### 4.4.2. Calcium

While dairy products are an important contributor of calcium to diets in many European populations, the intake of dairy foods varies widely across Europe, with per capita consumption being as low as 1.3 L/week (less than 200 mL/day) in some countries [43]. The prevalence of lactose malabsorption is high in some European populations, especially in Eastern Europe [44]. People also avoid dairy products for other health, ethical, or environmental reasons.

Most European countries have recommendations within their food-based dietary guidelines (FBDG) for the inclusion of dairy products, while some also include fortified plant-based alternatives in the guidelines. However, dairy is not a unique source of calcium [45]. Other calcium-rich plant foods consumed by Europeans include tofu, legumes, nuts and seeds, vegetables, and various calcium-fortified foods. The recommendations emphasize the importance of consuming fruits and vegetables in the diet, and some of these foods do contribute significantly to calcium intake (such as cruciferous and other leafy, green vegetables low in oxalates) and other nutrients (such as vitamin K, Mg, and K) and phytonutrients (such as flavonoids) that are essential to supporting bone health [46,47,48,49,50,51,52]. 

Calcium intake is of particular concern during adolescence and early adulthood when bone mass is accumulating. Given the sometimes poor quality of diets in adolescence, the availability of varied calcium sources, including fortified PBDs, may provide useful opportunities for improving the quality of the diet during this stage of growth. 

Over 80% of PBDs in Western European countries were found to be fortified with calcium to provide an amount comparable to dairy milk [53]. In a recent analysis, 76% of non-organic PBDs in Europe overall were calcium-fortified [14]. (European regulations for organic foods restrict the addition of vitamin and mineral fortification that is not legally required to be added) [54]. The bioavailability of the calcium in fortified products varies depending on the fortifying agent used but is generally similar to dairy milk. The main fortifying agents used are calcium carbonate and tricalcium phosphate. Calcium carbonate is absorbed from soya drinks at a rate comparable to the calcium in dairy milk [55]. The bioavailability of tricalcium phosphate is somewhat lower than the calcium in dairy, but the impact of this difference on calcium nutriture is unlikely to be significant [55,56]. Zhao et al. found that the amount of calcium absorbed from a soya drink fortified with tricalcium phosphate was only 3.6% less than the calcium absorbed from dairy milk [55]. For a soya drink fortified with 120 mg tricalcium phosphate/100 mL, this would translate to an 11 mg/serving difference in calcium absorbed compared to that absorbed from dairy milk.

#### 4.4.3. Vitamin B12 

Animal-based foods and B12-fortified plant foods are the only reliable sources of dietary vitamin B12 [57]. It is imperative that those who consume only plant-based diets consume fortified plant foods to obtain adequate vitamin B12. Otherwise, they will need to rely upon a regular dietary B12 supplement to avoid a B12 deficiency. Vitamin B12 deficiency is a common cause of macrocytic anaemia and has been implicated in a spectrum of neuropsychiatric disorders [58,59]. The deficiency can have serious long-term consequences.

For individuals who avoid animal foods altogether and who prefer not to take a B12 supplement, it is imperative that they consume two to three servings a day of foods fortified with vitamin B12 such as meat or dairy alternatives, yeast extracts, nutritional yeast flakes, and breakfast cereals [60]. Food manufacturers should provide such fortified foods readily available to the public. Supplementing foods with vitamin B12 may be vital to avoid vitamin B12 deficiencies, especially among those following a vegan diet. Non-dairy alternatives that are labelled as organic are not fortified in Europe, so it is important to advise individuals to select non-organic varieties fortified with vitamin B12. 

Vitamin B12 added to fortified foods does not require digestion before its absorption, since it is already in a free form. Vitamin B12 status is better maintained when consuming fortified foods or a B12 supplement than from meat, fish and other animal-based foods [61]. For elderly persons, with ageing-related impaired gastric secretions, their B12 status is improved by the regular use of a B12-fortified food such as a PBD [61,62]. 

Experts recommend that a mother following a 100% plant-based diet consume B12-fortified foods and ideally take a vitamin B12 supplement before and during pregnancy and during lactation. Low B12 intake and status during pregnancy or lactation have been linked to adverse maternal and perinatal health outcomes [63]. Vitamin B12-deficient pregnant women can lead to deficiency in the infant during the first few months of life [63,64]. 

The level of vitamin B12 in dairy milk can vary from about 0.5 to 1 mcg/serving (250 mL), although a wider range has been reported [65,66]. For comparison, PBDs in Europe typically contain about 1 mcg of B12 per serving [14]. In a 2021 study, about 40% of PBDs in selected countries of Western Europe were reported to be fortified with B12 [53], while a more recent analysis found that 45% of PBDs in Europe were fortified [30], while in another survey, 64% of non-organic PBDs and 44% of non-organic plant-based alternatives to yoghurt (PBAY) were fortified with B12 [14]. As dairy can be a significant source of B12, providing 17–40% of the total dietary B12 intake [67], increasing the degree of fortification to levels comparable to that of dairy, would be of benefit to consumers.

#### 4.4.4. Vitamin D 

Although vitamin D can be produced endogenously when skin is exposed to sunlight, many factors influence vitamin D status. Endogenous production can only occur at certain times of the year at certain latitudes and does not occur during autumn or winter months in high latitudes and consumers must become reliant on dietary sources and perhaps take supplements. The EFSA has established an adequate intake (AI) for vitamin D of 15 µg/day for children and adults based on an assumption of minimal endogenous synthesis [68]. 

Vitamin D has a limited distribution in foods and is found primarily as vitamin D3 (cholecalciferol) in fish oils, the flesh of fatty fish, and eggs from hens fed vitamin D [69]. Some wild mushrooms are a source of vitamin D2 (ergocalciferol) [70]. A vegan source of vitamin D3, isolated from lichen, is now available [71]. The intake of vitamin D from food alone varies among countries but is generally well below the AI in European populations. The mean intake of vitamin D was found to range from 1.1 μg/day in women in Spain to 8.2 μg/day in men in Finland [72]. Since an adequate intake of vitamin D is difficult to achieve by diet alone, dietary supplements of vitamin D are often recommended [71]. This vitamin D shortfall is aggravated by the fact that dairy is not routinely fortified with vitamin D across Europe, as it is in the US and Canada. 

Certain population groups have been identified as having a high risk for vitamin D deficiency and care should be taken to ensure adequate intakes, which may involve taking supplements. Such groups include the elderly (in which endogenous synthesis is reduced), institutionalized individuals who have little skin exposure to sunlight, pregnant women, and those living in countries at high latitudes.

The EFSA established a serum 25-hydroxyvitamin D (25(OH)D) concentration of 50 nmol/L as a suitable target value for establishing adequate vitamin D intake recommendations [68]. A serum 25(OH)D concentration of <50 nmol/L occurs in 40.4% of the population in Europe and in 26.0% of the general population in the United States [73,74,75]. The lower prevalence of poor vitamin D status in the United States may be explained in part by the availability of vitamin D-fortified foods. Dairy milk is routinely fortified with vitamin D in the United States; however, the fortification of dairy with vitamin D is uncommon in European countries, except for Finland, Sweden and Norway [14,76]. 

Concerns about inadequate vitamin D status in some European populations have led to calls for the fortification of a range of foods including not just dairy milk but country-specific staple foods, thereby ensuring adequate intakes and dietary diversity [77]. The practice of food fortification is a common and efficient approach to increasing daily nutrient intake and avoiding deficiencies. For example, the fortification of salt with iodine, and the fortification of flour with B vitamins and iron have done much to improve the nutritional status of populations [78,79]. 

PBDs fortified with vitamin D2 are currently available in most European countries, with over three-quarters being fortified with vitamin D2 [53], while about one-half of PBAY are fortified [14]. A meta-analysis of randomized clinical trials found that vitamin D2 is less effective than vitamin D3 at raising and maintaining 25(OH)D levels when provided in pharmacological doses such as 50,000 IU (1250 µg) or more [80]. However, when provided in daily doses of 1000 to 4000 IU (25–100 µg), which is more akin to dietary intakes, the difference was much smaller [80]. It is likely that vitamin D3 may be more effective when pharmacological doses are needed to reverse deficiency, but vitamin D2 is effective for maintaining 25(OH)D levels when consumed more regularly at doses typically found in fortified foods such as PBD.

#### 4.4.5. Iodine 

The World Health Organization promotes salt iodization as an effective means of ensuring adequate iodine intake [81]. As countries have adopted this policy, we see fewer populations encountering iodine deficiency [82,83,84]. However, in some countries in Europe, iodized salt is not widely used or the level of fortification is low [84], so suboptimal iodine status exists. There is a real need for implementing a standardized programme for the fortification of salt or other commonly consumed food product across Europe [85]. Aligned with this is the need to avoid excessive salt consumption and run counter to public health messaging to reduce salt intake for improved cardiovascular outcomes [86]. 

The iodine content of food varies widely depending on the soil levels and food processing. Dairy milk can significantly contribute to dietary iodine due to the cattle being fed iodine-supplemented feed and the milking sanitation procedures used on farms and in milk processing plants [87,88]. In countries where iodine-based disinfectants are not used, the iodine content of milk is much lower [89]. Iodine content is typically lower in organic milk [90,91] and in milk produced during the summer [92]. As a result, the iodine content of dairy milk is quite variable and values reported in food tables may not reflect the actual content [93,94]. 

Since milk is an important source of iodine in the UK and some other European countries, people who consume PBDs unfortified with iodine need to ensure they consume alternate iodine sources [91,95]. This also holds true for residents of those EU countries that are reluctant to permit iodine fortification due to health concerns of overconsumption. Some have called for plant-based dairy alternatives to be appropriately fortified with iodine at levels comparable to that of dairy milk [95]. In a recent Swiss study [96] the iodine content for eight different types of PBDs was reported to be 3–18% of the iodine level of dairy milk. In some European countries where it is permitted, manufacturers are slowly beginning to fortify PBDs with iodine; however, this is often at levels below those found in dairy milk. Only 12% of non-organic PBDs were reported to be fortified with iodine in a recent study [14]. For those at high risk of iodine deficiency due to their poor iodine intake, some recommend a daily iodine supplement of no more than 150 mcg [97].

#### 4.4.6. Riboflavin

Estimates of dietary intake of riboflavin in Europe, based upon surveys in nine countries [98], suggest that mean riboflavin intakes meet the recommendations established by the European Food Safety Authority (EFSA) [99] and exceed those established by both the Institute of Medicine (IOM) [100] and the WHO [101]. 

The main contributors of riboflavin to the diets of European populations are dairy products, grains and grain-based products, and meat products [99]. The enrichment of grains with B vitamins is less common in Europe than in the US, but even so, the riboflavin intake among European vegans appears to be adequate [102,103,104,105]. The riboflavin content of PBDs varies. Some PBDs are good sources, while other PBDs may provide much less riboflavin than dairy milk unless they are fortified [106,107]. In a recent study, just over one-half of non-organic PBDs were fortified with riboflavin with levels comparable to dairy, while only a few PBAY were fortified [14]. Good sources of riboflavin for those who do not consume dairy milk include leafy, green vegetables, soya foods, nutritional yeast, mushrooms, almonds, and fortified PBDs where available. 

### 4.5. Ultra-Processed Food Classification

In recent years, a new approach to food classification has been developed, which is based on the degree of processing rather than on nutrient composition. The most common classification system used is NOVA. This assigns foods into one of four categories: category 1 is minimally/unprocessed foods (e.g., milk, plain yoghurt); category 2 includes processed culinary ingredients (e.g., butter, oils, sugar); category 3 includes processed foods (e.g., canned vegetables, cured meats); and category 4 includes ultra-processed foods (UPFs) which are defined as “ready-to-eat, industrially formulated foods” and include chocolate, ice cream, biscuits, and fruit yoghurts [108]. The foods in category 4 are often characterized as having high energy density, high glycaemic index, and low satiety [109,110].

A number of epidemiological studies have reported associations between the consumption of UPFs and the risk of obesity, metabolic syndrome, colon cancer, and mortality [111,112,113], but the use of this system in nutritional epidemiology studies has been challenged. Reports of contradictory findings have led to some questioning the validity of NOVA [114,115,116]. A healthy eating pattern is defined by NOVA in terms of the degree of food processing rather than by the nutritional content of the food [116].

The definitions of UPFs are inconsistent between various classification systems [116] and, furthermore, criticism has been expressed about the difficulty of distinguishing between ultra-processed products with differing nutritional qualities [117,118]. This is particularly relevant for fortified PBDAs, which are usually classified as UPFs because of added ingredients including additives such as stabilizers and emulsifiers and micronutrients, whereas their dairy counterparts (including milk and plain yoghurts) are considered unprocessed or minimally processed [119,120]. Despite this, recent studies demonstrate that PBDAs may exhibit similar or better nutritional quality than their dairy counterparts. For example, the energy density of soya-based drinks is lower than that of dairy milk, both whole and low fat, and they are lower in saturated fat and similar in protein content [121]. Furthermore, they have been shown to have a low glycaemic index [122]. Other PBDAs also score well in terms of low sodium content, saturated fat and calories [53] and additionally contain a range of naturally occurring bioactive components, such as polyphenols, with potential health benefits [123,124]. Soya-based drinks also have substantial levels of folate and some trace minerals compared to dairy milk [125]. Importantly, in contrast to dairy products, PBDAs have a substantially lower environmental impact, as seen above, in terms of greenhouse gas emissions, land use, eutrophication, and water use [11].

Food processing plays an important role in food security and safety, but some processes can also lead to products high in salt, sugars, and saturated fats [126]. However, it is clear that classifying PBDAs as UPFs, with the implied adverse nutritional and health associations, is inconsistent with current findings regarding the nutritional quality of such products and may discourage people from transitioning to a plant-based diet with all its health and environmental advantages.

There have been calls in the scientific press to revise the NOVA criteria so that healthy plant proteins, natural stabilizers, and vitamin and mineral mixes do not constitute ultra-processing and that the NOVA guide should consider the fat, salt, and sugar content of the food [119] In the UK, the influential consumer group Which? has pointed out some of the inconsistencies in the definitions of processed and ultra-processed foods. Rather than using these terms to decide whether a food is healthy or not, food companies have provided “traffic light” information on food labels for consumers to make appropriate choices [127].

### 4.6. Factors Influencing Change towards Plant-Based Dairy Alternatives

Taste, cost, health, availability, convenience, and ethical and environmental concerns are important influences on food choices as consumers search for healthier and more sustainable diets [128,129,130,131]. These motivations must be addressed to enhance the popularity of PBDAs. Because these PBDAs are designed to mimic milk-based products, they can conveniently be substituted without the consumer needing to change their habitual eating patterns. For example, the British Dietetic Association’s One Blue Dot^®^ resource on meal swaps includes making breakfast more sustainable by using a fortified PBD rather than milk with breakfast cereal [7]. 

The increased availability of PBDAs on supermarket shelves, usually alongside the dairy version, indicates that PBDAs are no longer considered a niche market [9]. Not only does this location of PBDAs make their purchase more convenient for would-be consumers, but the location could also subconsciously increase purchasing by “nudging” consumers at the point of choice by acting as a cue, reducing the effort required and changing the perception of social norms [132]. A lack of availability in local grocery stores, and in pre-school settings, as well as a lack of products marketed towards children, have been identified as barriers to increasing the consumption of PBDAs [133].

The sensory properties (especially taste) of foods and drinks are one of the most important motivations for food choice [134]. Those who are already consumers of PBDAs appraise the sensory properties of the products positively [135,136]; however, taste and taste perception remains one of the biggest hurdles to overcome for non-consumers. Familiarity with foods is an important positive influence on food preferences and food choices across the lifespan [137]. Food neophobia (avoidance of the ingestion of novel foods) can be reduced through repeated tasting (exposure) of the novel food, which in turn increases the consumption of the food [137]. Increasing consumers’ opportunities for tasting PBDAs will likely increase preference and increase intake. 

Awareness of the environmental impact of prioritizing plant-based proteins is relatively low compared to other sustainable diet recommendations [138,139]. Greater awareness and the perceived importance of consuming healthy and sustainable diets are associated with increased willingness to adopt sustainable dietary recommendations and increased purchasing of plant-based foods, including PBDAs [138,140,141,142,143]. 

Although the higher cost of PBDAs [144] could be a barrier to consumption [133,142,143], the continued growth in the market and the development of new PBDAs driven by increasing demand from consumers suggests that increasing the consumption of PBDAs is a potential driver for shifting population intakes towards more healthy and sustainable plant-based diets [145].

In our analysis, we observed the average retail price of the leading brand of fortified soya drink among the five leading retail stores in the UK to be about one-third more expensive (an additional 47 p/L) than the in-house/store brand (private label) soya drink and 45% more expensive than the dairy semi-skimmed milk. The price differential between 1 L of the private brand of soya and the dairy milk equivalent was a mere six pence. As more retailers produce their own private-label versions, the price differential with dairy is expected to shrink. 

As mentioned earlier, PBDAs are often chosen by consumers for health reasons. It has been reported elsewhere that PB drinks that are not based on coconut have low levels of saturated fat. They also contain no cholesterol and provide a measurable amount of unsaturated and dietary fibre [53]. Some drinks based on walnuts or seeds (flax, hemp) have measurable amounts of omega-3 fat. PB drinks such as soy are also a rich source of health-promoting flavonoids, phytosterols, and other phytochemicals, making them useful foods in the fight against heart disease and other chronic diseases [146,147,148,149,150].

## 5. Conclusions

PBDs have a substantially lower environmental footprint than dairy milk. The production of plant-based dairy alternatives uses markedly fewer natural resources, such as land and water, and greenhouse gas emissions are considerably lower in their production. With present concerns about climate change, planetary health, and the need to consume a more sustainable diet, PBDAs will continue to be popular among environmentally minded consumers in addition to those who are dairy intolerant. 

PBDs made from soya and pea have ample protein with regard to both quantity and quality. Fortified non-organic varieties, provide levels of calcium and vitamin D comparable to dairy milk. The consumption of PBDAs fortified with nutrients such as calcium, and for some countries iodine and vitamins B2 and B12, would ensure nutritional adequacy among healthy European populations transitioning to a more plant-based sustainable eating pattern. Fortified PBDs with these critical nutrients can provide nutrients in quantities comparable to dairy milk [14].

When one serving of dairy milk in the EAT Lancet’s Planetary Health Diet model is replaced by a serving of a fortified PBD, the overall nutrient content of the diet experiences minimal changes while significantly reducing the environmental impact. Calcium levels remain unchanged, while vitamin A and D levels increase. Despite the protein level of the diet with the substituted oat drink falling by 7%, the total protein intake still exceeds the DRI for protein. 

Attempts to combine a nutrient density score with an environmental index to prioritize foods for a more sustainable diet have encountered several challenges. The efforts to merge such data can easily lead to a misclassification of food when the full nutrition profile is not considered or when only a selection of environmental factors is taken into account. The categorization of PBDAs as UPFs implies that they have adverse nutritional and health attributes. However, such a classification is inappropriate given the demonstrable nutritional quality and lower environmental impact of these PBDAs. 

Imperative to the growth of PBDAs is the willingness to adopt sustainable dietary recommendations and give the assurance that these drinks support a healthy and environmentally sustainable diet. Our observations highlight that substituting dairy milk with a fortified PBDA does not compromise the overall nutritional quality of the diet while significantly reducing the environmental footprint.

## Figures and Tables

**Table 1:** The adapted EAT Lancet Planetary Health Diet (EAT PHD) nutritional values using a European food database compared to the original EAT Lancet analysis using a US food database.

	Original EAT PHD Analysis [8]	EU EAT PHD with 250 mL Low-Fat Dairy Milk ^1^		
		Nutrients	% Difference ^2^	% DRV ^3^
kJ	10,500	9946	−1%	104%
Kcal	2500	2370	−1%	104%
Fat g	105.6	99.4	−4%	112%
Saturated Fat g	22.7	21.3	−9%	106% ^4^
Carbohydrate g	317.3	277.2	0%	97%
Total Sugars g	NA	83.4	NA	146%
Fibre g	42.9	36.7	0%	147%
Protein g	90.1	89.2	−1%	154%
Salt g	not > 5.75	0.61	NA	10%
Calcium mg	718	729	2%	73%
Iodine mcg	NA	84.5	NA	56%
Vit A (REA) mcg	1068	1186	−6%	158%
Vit D mcg	4.87	2.17	−60%	14%
Riboflavin (B2) mg	1.70	1.45	2%	90%
Vit B12 mcg	2.30	2.91	−1%	73%

^1^ The EU Baseline EAT PHD diet analysis reflects the original EAT Lancet Planetary Health Diet recommended foods and specified quantities with one adaptation—the 250 mL daily full-fat dairy milk allowance was switched to 250 mL low (1.2–1.8%)-fat dairy milk. ^2^ % difference from the original EAT PHD nutritional analysis. ^3^ % DRV = the % of EU adult dietary reference values. ^4^ Based on the daily 20 g reference intake set by the labelling regulation EU No 1169/2011 on the provision of food information to consumers. The EU has not set a DRV for saturated fat and instead recommends keeping it as low as possible. REA = Retinol Reactive Equivalents. NA = not available; total sugars, iodine values, and absolute salt values are not specified by the EAT Lancet publication.

**Table 2:** Nutritional comparison between the Baseline EU EAT Lancet PHD and switching the daily 250 mL low-fat dairy allowance with fortified plant-based drinks.

	Baseline EU EAT PHD with 250 mL Low-Fat Dairy Milk ^1^		Replacing the 250 mL Low-Fat Diary Milk in the Baseline EU EAT PHD with a Fortified Plant-Based Drink					
			250 mL Fortified Soya Drink			250 mL Fortified Oat Drink		
	Nutrients	% DRV ^3^	Nutrients	% Difference ^2^	% DRV ^3^	Nutrients	% Difference ^2^	% DRV ^3^
kJ	9946	104%	9860	−1%	103%	9913	0%	103%
Kcal	2370	104%	2350	−1%	103%	2362	0%	104%
Fat g	99.4	112%	100	1%	113%	99.3	0%	112%
Sat. Fat g	21.3	84%4	19.5	−8%	77%^4^	19.3	−9%	76% ^4^
Carbohydrate g	277.2	97%	271.5	−2%	95%	281.7	2%	99%
Total Sugars g	83.4	146%	76.6	−8%	134%	79.9	−4%	140%
Fibre g	36.7	147%	37.9	3%	152%	38.7	5%	155%
Protein g	89.2	154%	88.4	−1%	152%	82.9	−7%	143%
Salt	0.61	10%	0.61	0%	10%	0.61	0%	10%
Calcium mg	729	73%	729	0%	73%	729	0%	73%
Iodine ug	84.5	56%	110.5	31%	74%	110.5	31%	74%
Vit A (REA) mcg	1186	158%	1151	−3%	153%	1151	−3%	153%
Vit D ug	2.17	14%	4.02	85%	27%	4.02	85%	27%
Riboflavin (B2) mg	1.45	90%	1.52	5%	95%	1.52	5%	95%
Vitamin B12 mcg	2.91	73%	2.76	−5%	69%	2.76	−5%	69%

^1^ The baseline EU EAT PHD diet analysis reflects the original EAT Lancet Planetary Health Diet recommended foods and specified quantities with one adaptation—the 250 mL daily full-fat dairy milk allowance was switched to 250 mL low (1.2–1.8%)-fat dairy milk. ^2^ % difference from the baseline EU EAT PHD. ^3^ % DRV = the % of EU adult dietary reference values. ^4^ Based on the daily 20 g reference intake set by the labelling regulation EU No 1169/2011 on the provision of food information to consumers. The EU has not set a DRV for saturated fat and instead recommends keeping it as low as possible. REA = Retinol Reactive Equivalents. NA = not available; total sugars, iodine values, and absolute salt values are not specified by the EAT Lancet publication.

## Supplementary Materials

The following supporting information can be downloaded at: https://www.mdpi.com/article/10.3390/nu15153393/s1, Ingredient Sources and Cost Analysis.
