# Supplementary material for: Plant-Based Dairy Alternatives Contribute to a Healthy and Sustainable Diet
Source: Nutrients. 2023 Jul 30;15(15):3393. doi: 10.3390/nu15153393 (PMC10421454; doi:10.3390/nu15153393)
Supplement: Supplementary file 1 [file nutrients-15-03393-s001.zip › nutrients-2507294-supplementary.pdf]

Identifying Ingredient Sources for PBD

It was assumed that all PBDs consumed in the EU are also produced in European countries. It was challenging to retrieve trade data for PBDAs on a European scale to account for the grains and legumes grown outside of Europe that were processed for the PBDA market. However, with a small percentage of the vegan population in Europe in 2020 (3.2%) who are most likely to consume PBDAs, it is fair to assume that EU crops were used. Many PBDAs highlight on their packaging that a European crop was processed with locally sourced produce, assumed to be more sustainable.

Comparative Analysis of the Cost of Soya Drinks and Dairy Milk in the UK.

Drink prices in UK.xlsx - Excel

Product	Soya Chilled Drink	Soya chilled drink	Dairy semi-skimmed milk	Plain Soya PBAY (chilled)	Plain Soya PBAY (chilled)	Dairy Low Fat Natural Yogurt	PRICE DIFFERENCE SOYA DRINK vs DAIRY SEMI-SKIMMED M			
Brand	Alpro	Retail/ private label	Retail/ private label	Alpro	Retail/ private label**	Retail/ private label		1L Alpro soya drink vs 1L Retail soya drink	1L Alpro soya drink vs 1L dairy semi-skimmed milk	1L Retail/Private Label soya drink vs 1L dairy semi-skimmed milk
Volume	1L	1L	1.136L	500g	500g	500g	£ DIFFERENCE	£0.47	£0.55	£0.06
Morrisons	£1.50	£1.35	£1.20	£2.10		£1.09	% difference	36%	45%	5%
Asdas	£2.00	£1.35	£1.20	£2.10	£1.45	£1.10				
Sainsbury's	£1.95	£1.25	£1.20	£2.10		£0.70				
Waitrose	£2.00	£1.30	£1.30	£2.10		£0.75				
Tescos	£2.10	£1.25	£1.25	£2.10	£1.45	£1.10				
Average	£1.91	£1.30	£1.23	£2.10	£1.45	£0.95				
